# Sex on Steroids: How Brassinosteroids Shape Reproductive Development in Flowering Plants

**DOI:** 10.1093/pcp/pcae050

**Published:** 2024-04-26

**Authors:** Rita B Lima, Duarte D Figueiredo

**Affiliations:** Plant Reproductive Biology and Epigenetics, Max Planck Institute of Molecular Plant Physiology, Potsdam Science Park, Am Mühlenberg 1, Potsdam 14476, Germany; Plant Reproductive Biology and Epigenetics, Max Planck Institute of Molecular Plant Physiology, Potsdam Science Park, Am Mühlenberg 1, Potsdam 14476, Germany

**Keywords:** Brassinosteroids, Fruits, Gametes, Reproduction, Seeds, Yield

## Abstract

**Since the discovery of brassinolide in the pollen of rapeseed, brassinosteroids (BRs) have consistently been associated with reproductive traits. However, compared to what is known for how BRs shape vegetative development, the understanding of how these hormones regulate reproductive traits is comparatively still lacking. Nevertheless, there is now considerable evidence that BRs regulate almost all aspects of reproduction, from ovule and pollen formation to seed and fruit development. Here, we review the current body of knowledge on how BRs regulate reproductive processes in plants and what is known about how these pathways are transduced at the molecular level. We also discuss how the manipulation of BR biosynthesis and signaling can be a promising avenue for improving crop traits that rely on efficient reproduction. We thus propose that BRs hold an untapped potential for plant breeding, which could contribute to attaining food security in the coming years**.

## Reproductive Development in Flowering Plants

**Fig. 1:**
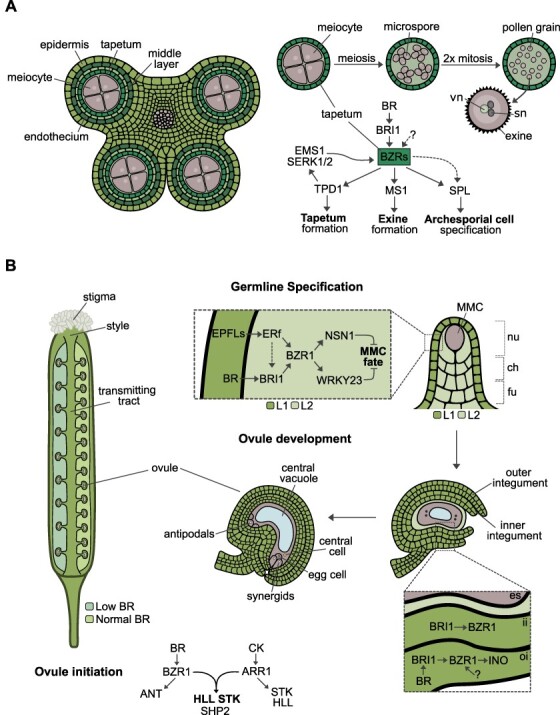
BR functions in male and female fertility. (A) BRs regulate male sterility. Schematic representation of anther and pollen grain development in the model plant *Arabidopsis*. BRs modulate archesporial cell specification through *SPL* in a *BRI1*-dependent (solid arrow) and independent manner (dashed arrows). The BZRs are expressed in the tapetal cells, where they bind to the promoter of genes involved in pollen and tapetum development and activate their expression. BZRs also function downstream of the TPD1-EMS1-SERK1/2 module to regulate tapetum formation. vn, vegetative nucleus; sn, sperm nucleus. (B) Roles of BRs in female reproductive development. Illustration showing the reproductive tract of *Arabidopsis* and different stages of ovule development. BRs and CKs promote ovule initiation. BZR1 and ARR1 together target ovule initiation genes such as *STK* and *HLL*, which are known BR and CK targets, or genes that are only expressed in the presence of both hormones, like *SHP2*. BRs prevent somatic cells surrounding the MMC from acquiring germline fate in a *BRI1*-dependent and *BRI1*-independent (EPFL-ERf) manner. BRs then regulate outer integument development via BZR1-mediated activation of *INO*. nu, nucellus; ch, chalaza; fu, funiculus; es, embryo sac; ii, inner integument; oi, outer integument.

**Fig. 2:**
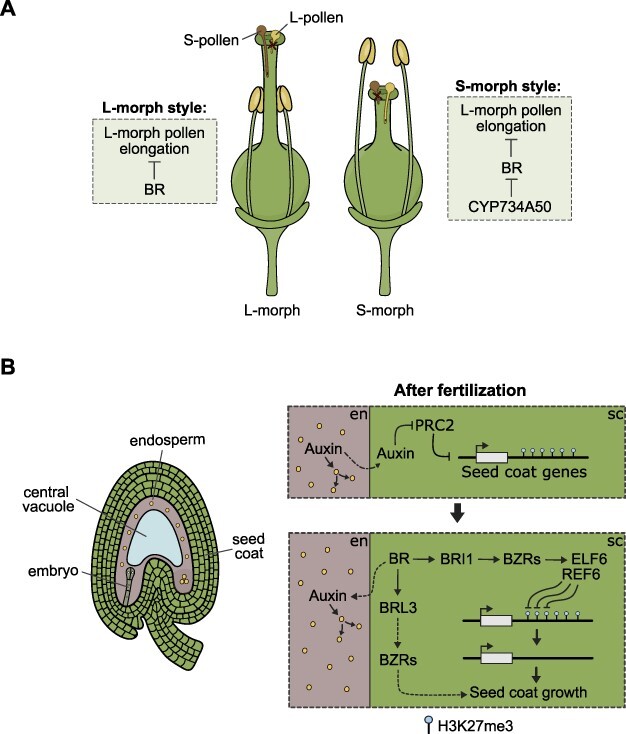
BR roles in hybridization barriers and seed development. (A) BR activity regulates pollen germination and elongation in incompatible mating systems. Schematic of the mating types observed in heterostylous *Primula*. BRs repress the elongation of L-pollen tubes in the styles of L-morphs, while CYP734A50 promotes BR degradation and consequent L-pollen tube growth in S-morphs styles, thereby preventing self-fertilization. (B) BRs regulate seed development. Illustration of a developing *Arabidopsis* seed. Upon fertilization, endosperm-derived auxin (dotted-dashed arrow) blocks the activity of the sporophytic PRC2. The expression of genes involved in seed coat development requires the active removal of the H3K27me3 marks previously deposited by the PRC2. This removal is likely mediated by BR signaling via BRI1, as BZRs have been shown to recruit the histone demethylases REF6 and ELF6. In addition, BR signaling via BRL3 also promotes seed coat growth, possibly via BZRs, independent of BRI1 (dashed arrows). BRs non-cell autonomously regulate endosperm proliferation by promoting seed coat expansion and regulating auxin activity through a not yet identified mechanism. en, endosperm; sc, seed coat.

Reproductive structures of angiosperms are found within the flower. The two innermost whorls are formed by the androecium and the gynoecium, which are the male and female reproductive organs, respectively. In the androecium, also known as stamens, a long filament supports the anther, where the pollen grains, or male gametophytes, are formed (**Fig. 1A**) (Gómez et al. 2015). The gynoecium or carpel comprises the stigma, style and ovary, which contains the ovules (**Fig. 1B**) (Herrera-Ubaldo and De Folter 2022). The ovule is defined by the female gametophyte (FG), or embryo sac, which is surrounded by sporophytic integuments (**Fig. 1B**) (Rudall 2021). The mature pollen grain typically consists of two sperm cells engulfed in a vegetative cell (**Fig. 1A**). While there is some variation in the structure of the mature embryo sac, depending on the species of origin, here we focus on the *Polygonum* type, which is characteristic of most angiosperms (Yadegari 2004). This type of megagametophyte is formed by seven cells: three antipodals, two synergids, one egg cell and one central cell (**Fig. 1B**). The latter results from the fusion of two polar nuclei, meaning that it is diploid, in contrast to all other gametophytic cells, which are haploid (Yadegari 2004). Once the pollen grain lands on the stigma, the vegetative cell elongates giving rise to the pollen tube, which grows through the female reproductive tissues reaching the ovule (**Fig. 2A**). There, the sperm nuclei are released into the embryo sac where one fertilizes the egg cell giving rise to the embryo and the other fuses with the central cell originating the nourishing endosperm (**Fig. 2B**) (Bleckmann et al. 2014). Fertilization products develop embedded in the maternal seed coat tissues that arise from the differentiation of the integuments (Haughn and Chaudhury 2005). In most angiosperms, seed development takes place within a fruit, which is derived from the ovary tissues, and these structures develop in a synchronous manner (Dong and Østergaard 2019).

Evidence pointing to the involvement of brassinosteroids (BRs) in reproductive development emerged decades ago with the discovery of BRs in the pollen of *Brassica napus* (rapeseed) (Mitchell et al. 1970, Grove et al. 1979). In fact, pollen is one of the richest sources of endogenous BRs and the reduced male fertility observed in mutants with impaired BR biosynthesis or signaling reinforces the importance of this hormone in the male reproductive tissues (Clouse et al. 1996, Li et al. 1996, Szekeres et al. 1996, Clouse and Sasse 1998). Since its isolation, BR functions have also been implicated in female reproductive development and in post-fertilization processes. Even so, in contrast to what is known for vegetative development, studies elucidating how BRs regulate fertility at the molecular level are still lacking. Therefore, in this review, we aim to discuss the current knowledge on the roles of BRs during reproductive development and convey the different reproductive processes in which the function of BRs could be further investigated. We not only refer mostly to what is known in the model species *Arabidopsis thaliana* (*Arabidopsis*) but also discuss what is known in other species, including crops. In **Table 1**, we compiled a list of reproductive phenotypes described in the literature that characterize various BR mutant alleles.

**Table 1:** Phenotypes of BR mutants in reproductive tissues

Mutant	Phenotypes	References
***Arabidopsis thaliana***		
*bri1-116*	Mild to severe outer integuments growth arrest Reduced ovule curvature Abnormal FGs Multiple enlarged MMC-like cells Reduced meiocyte and microspore number Large and vacuolated microspores Abnormal tapetum	Jia et al. (2020) Cai et al. (2022) Ye et al. (2010)
*bri1-5*	Slight outer integument growth defect Reduced ovule and seed number	Jia et al. (2020) Huang et al. (2013)
*bri1-6*	Smaller and round seed coats Reduced endosperm proliferation	Pankaj et al. (2023) Lima et al. (2023)
*bri1-10*	Reduced pollen tube growth	Vogler et al. (2014)
*bri1 brl1 brl3* (*bri-t*)	Mild to severe outer integument growth arrest	Jia et al. (2020)
*bzr-h*	Severe outer integument growth arrest Absence of loculi in anthers	Jia et al. (2020) Chen et al. (2019a)
*qui-1*	Multiple enlarged MMC-like cells Collapsed embryo sacs Anthers with abnormal tapetal cells Development arrested at the meiocyte stage	Cai et al. (2022) Chen et al. (2019a)
*bin2-1*	Precocious vacuole formation in early embryo sac development Increase ovule and seed number	Cai et al. (2023) Huang et al. (2013)
*br6ox1 br6ox2*	Reduced endosperm proliferation	Lima et al. (2023)
*br6ox2 (cyp85a2-2)*	Short anthers	Kim et al. (2005b)
*bin2-3 bil1 bil2*	Arrested embryo sac prior to nuclear division Arrested embryo sac due to failure in large vacuole formation Degenerated embryo sacs in late development	Hu et al. (2023)
*cpd*	Supernumerary MMC-like cells Reduced meiocyte and microspore number Large and vacuolated microspores Abnormal tapetum Reduced pollen tube growth	Cai et al. (2022) Ye et al. (2010) Vogler et al. (2014)
*cpd-91*	Reduced endosperm proliferation	Lima et al. (2023)
*det2-1*	Slight outer integument growth defect	Jia et al. (2020)
*det2-1*	Reduced ovule and seed number Reduced endosperm proliferation Smaller and round seed coat	Huang et al. (2013) Lima et al. (2023) Jiang et al. (2013); Pankaj et al. (2023)
*det2-9*	Supernumerary MMC-like cells	Cai et al. (2022)
*dwf4-44*	Defective outer integuments	Lima et al. (2023)
*dwf4*	Multiple enlarged MMC-like cells	Cai et al. (2022)
*dwarf5-1*	Smaller and round seeds	Choe et al. (2000)
*br6ox2*	Slight reduction in outer integument growth Absence of FGs Supernumerary MMC-like cells	Nole-Wilson et al. (2010) Cai et al. (2022)
*brl3*	Smaller seed coat	Pankaj et al. (2023)
*bzr1-D*	Defective FGs with persistent nucellus and two nuclei instead of a single central cell at maturity Increased ovule and seed number	Lima et al. (2023) Huang et al. (2013)
*shrink1-D*	Smaller and round seeds	Takahashi et al. (2005)
***Primula forbesii***		
*cyp734a50*	Increased anther size Loss of SI	Huu et al. (2022)
***Oryza sativa***		
*dwarf1*	Shorter grains	Hong et al. (2005)
*dwarf11*	Shorter grains	Tanabe et al. (2005)
*Osgata6*	Larger grains	Zhang et al. (2022)
***Pisum sativum***		
*lk*	Small and irregularly shaped seeds	Nomura et al. (2007)
***Solanum lycopersicum***		
*dwf4*	Fewer ovules	Barro-Trastoy et al. (2020a)

## BR Biosynthesis and Signaling Pathways

**Fig. 3:**
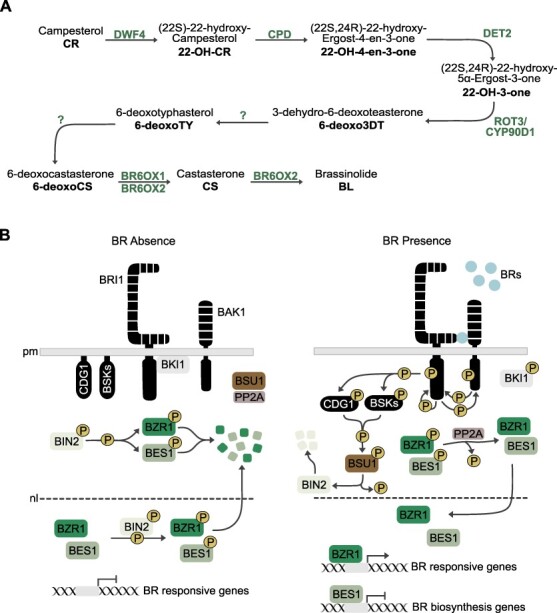
BR biosynthesis and signaling pathways. (A) Linear BR biosynthetic pathway and the enzymes catalyzing each step. (B) Simplified representation of the BR signaling pathway. In the absence of BR (left-hand panel), BRI1 is inhibited by BRI1 KINASE INHIBITOR 1 (BKI1). BIN2 phosphorylates BZR1 and BES1, which are degraded by the proteasome. In the presence of BR (right-hand side), BRI1 binds to the co-receptor BAK1. BKI1 dissociates from BRI1, allowing BRI1 and BAK1 transphosphorylation and consequent activation. This initiates a cascade of phosphorylation and dephosphorylation, which leads to BZR1 and BES1 dephosphorylation by PP2A. BZR1 and BES1 move to the nucleus where they activate genes involved in BR response and repress genes that participate in BR biosynthesis.

BRs are plant steroid hormones that are involved in numerous developmental processes, such as vascular differentiation, photomorphogenesis, stress responses and reproduction (Ye et al. 2010, Clouse 2011a, Planas-Riverola et al. 2019, Nolan et al. 2020). Since the purification of brassinolide (BL) more than 40 years ago was from the pollen of rapeseed, more than 70 compounds have been identified belonging to this class of hormones (Grove et al. 1979, Zhao and Li 2012). Even so, BL, the final product of the BR biosynthetic pathway, is still the most bioactive BR identified to date (Clouse 2011b). The biosynthesis of BR starts from campesterol (CR), which undergoes several rounds of enzymatic reactions to produce the ultimate product, BL. These reactions are catalyzed by enzymes that can use several substrates to produce distinct intermediates within the pathway, leading to a grid-like biosynthetic pathway. However, through extensive experimentation, it became possible to organize the biosynthetic route in a linear fashion (Clouse 2011b, Zhao and Li 2012). **Fig. 3A** shows this biosynthetic route. According to the linear pathway, CR is first hydroxylated into (22*S*)-22-hydroxy-campesterol (22-OH-CR) by the enzyme CYP90B1 encoded by *DWARF4* (*DWF4*), which is posteriorly converted into (22*S*,24*R*)-22-hydroxy-ergost-4-en-3-one (22-OH-4-en-3-one) by the *CONSTITUTIVE PHOTOMORPHOGENIC DWARF* (*CPD*)–encoded enzyme CPY90A1 (Choe et al. 1998, Ohnishi et al. 2012). This is followed by the conversion of 22-OH-4-en-3-one to (22*S*,24*R*)-22-hydroxy-5α-ergost-3-one (22-OH-3-one) catalyzed by a 5α-reductase encoded by the gene *DE-ETIOLATED2* (*DET2*) (Chory et al. 1991, Li et al. 1996, Noguchi et al. 1999). Then, the C-23 hydroxylases CYP90C1, encoded by *ROTUNDIFOLIA3* (*ROT3*), and CYP90D1 act redundantly to convert 22-OH-3-one into 3-dehydro-6-deoxoteasterone (Kim et al. 1998; Kim 2005, Ohnishi et al. 2006b). This latest intermediate is converted to 6-deoxotyphasterol (6-deoxoTY), which in turn is converted into 6-deoxocastasterone (6-deoxoCS), by enzymes that remain to be identified. Then, the C-6 oxidases CYP85A1 and CYP85A2, encoded by *BR6OX1* and *BR6OX2*, respectively, convert 6-deoxoCS into castasterone (CS). The BR6OX2-encoded enzyme has the additional function of oxidizing CS to BL, thereby catalyzing the last step of the pathway (Noguchi et al. 2000, Kwon et al. 2005, Nomura et al. 2005, Kim et al. 2005).

While animal steroid hormones can bind to nuclear receptors to modulate gene expression, BRs bind to receptors that localize to the plasma membrane. A simplified scheme of the BR signaling pathway can be found in **Fig. 3B**. Upon synthesis in the endoplasmic reticulum (ER), BL is transported to the apoplast where, at the cell surface, it encounters the leucine-rich repeat receptor kinases BRASSINOSTEROID INSENSITIVE1 (BRI1), BRI-LIKE1 (BRL1) and BRI-LIKE3 (BRL3) (Li and Chory 1997, Caño-Delgado et al. 2004). The binding of BL to BRI1 results in a structural rearrangement of the receptor, which allows its interaction with the co-receptor EMBRYOGENESIS RECEPTOR KINASE3/BRI1-ASSOCIATED KINASE1 (SERK3/BAK1) (Li et al. 2002, Hothorn et al. 2011, She et al. 2011). The interaction between BRI1 and BAK1 initiates progressive transphosphorylation between the two receptors, rendering a fully activated BRI1 that proceeds to phosphorylate the membrane-anchored BR SIGNALING KINASES (BSKs) and CONSTITUTIVE DIFFERENTIAL GROWTH1 (CDG1) (Tang et al. 2008, Kim et al. 2011, Ren et al. 2019). The BRI1-mediated phosphorylation of BSKs and CDG1 results in their release from the membrane and activation of the phosphatase BRI1-SUPPRESSOR1 (BSU1), which in turn dephosphorylates the GSK3-like kinase BRASSINOSTEROID-INSENSITIVE 2 (BIN2), a negative regulator of BR signaling (Li and Nam 2002). In the absence of BL, BIN2 represses BRASSINAZOLE RESISTANT1 (BZR1) and BRI1-EMS-SUPRESSOR1 (BES1), the main transcription factors (TFs) involved in BR responses (Wang et al. 2002, Yin et al. 2002). BZR1 and BES1 belong to a family of atypical basic helix-loop-helix (bHLH) TFs that also includes the BES1/BZR1 homologs BEH1, BEH2, BEH3 and BEH4, which play redundant roles in BR-mediated gene expression (Chen et al. 2019a, Kim et al. 2023). The phosphorylation of BZR1 and BES1 by BIN2 targets them for proteasomal degradation and/or enhances their translocation from the nucleus to the cytoplasm mediated by 14-3-3 proteins (He et al. 2002, Yin et al. 2002, Vert and Chory 2006, Gampala et al. 2007, Ryu et al. 2007). The dephosphorylation of BIN2 by BSU1 leads to its proteasomal degradation promoted by the E3 ubiquitin ligase KINK SUPPRESSED IN BZR1-1D and consequent release of BZR1 and BES1 from BIN2-mediated inhibition (Zhu et al. 2017). This is followed by BZR1 and BES1 dephosphorylation by PROTEIN PHOSPHATASE 2A (PP2A) and accumulation in the nucleus, where they regulate the expression of numerous BR-responsive genes (Sun et al. 2010, Tang et al. 2011, Yu et al. 2011). Therefore, the binding of BL to the receptor sets the beginning of a cascade of phosphorylation and dephosphorylation, which culminates in the activation of the key TFs, BZR1 and BES1. The establishment of BZR1 and BES1 gene networks not only revealed the extensive scope of BR-regulated processes but also demonstrated the vast interaction with other hormonal pathways throughout plant development.

## BR Functions during Reproductive Organ Formation and Gametogenesis

### Roles of BR in anther and pollen development

The development of the anther initiates from three distinct cell layers of the floral meristem: the epidermis (L1), the subepidermis (L2) and the core (L3) (Jenik and Irish 2000). The internal growth of the anther occurs mostly due to cell divisions of a single L2 cell, the archesporial cell, which undergoes a first round of division to originate a primary parietal cell (PPC) and a primary sporogenous cell (PSC) (Canales et al. 2002, Scott et al. 2004). The PPC divides multiple times giving rise to three cell layers that surround the meiocytes, the product of PSC divisions. Therefore, from inwards to outwards, an anther locule is formed by the meiocytes encapsulated by the PPC-derived tapetum, middle layer and endothecium, and the L1 (**Fig. 1A**) (Gómez et al. 2015). Within the layered structure, the meiocytes undergo meiosis and originate a tetrad of haploid microspores (**Fig. 1A**) (Owen and Makaroff 1995). Upon release to the anther locule, the microspores divide twice mitotically to originate the pollen grain (**Fig. 1A**) (Owen and Makaroff 1995). Reduced male fertility has been described for mutants impaired in BR biosynthesis and perception. Detailed analysis of the anthers and pollen of *cpd* and *bri1-116* mutants of *Arabidopsis* revealed a reduction in the number of meiocytes and consequently a dramatic decrease in microspore number (Ye et al. 2010). These microspores were also larger, more vacuolated and surrounded by abnormal tapetal cells (Ye et al. 2010). In addition, the release of *cpd* and *bri1-116* pollen after anther dehiscence was also reduced, a phenotype that was attributed to the abnormal structure of the exine pattern, which led to pollen adhesion to the anther inner wall (Ye et al. 2010). Real-time qRT-PCR analysis and chromatin immunoprecipitation (ChIP) experiments demonstrated that BES1 targets the promoter of many genes involved in anther and pollen development, thereby regulating their expression (Ye et al. 2010). Among the genes differentially regulated in *cpd* and *bri1-116*, floral buds were *SPOROCYTELESS* (*SPL*), encoding a MADS-box TF that is necessary for the specification of archesporial cells, *EXCESS MICROSPOROCYTES1* (*EMS1*), encoding a leucine-rich repeat transmembrane protein kinase, which is required for tapetum formation, and *MALE STERILITY1* (*MS1*), which encodes a TF involved in exine formation (Yang et al. 1999, Wilson et al. 2001, Canales et al. 2002, Zhao et al. 2002, Ito et al. 2007, Ye et al. 2010). In fact, all members of the BZR family of TFs have been linked to male fertility. But, curiously, the BZR-mediated regulation of anther and pollen development seems to be in part independent of BR (Chen et al. 2019; Chen et al. 2019). Two independent studies showed that the high-order mutants *bes1-1 bzr1-1 beh1-1 bzr3-1 bzr4-1* (*qui-1*) and *bes1-4g bzr1-4a beh1 beh2-4a beh3 beh4* (*bzr-h*) are sterile (Chen et al. 2019; Chen et al. 2019). However, while in the *qui-1* mutant, the sterility was caused by anthers with abnormal tapeta and meiocytes that arrested development, in the *bzr-h* mutant the anthers lacked loculi (Chen et al. 2019; Chen et al. 2019). Instead of the well-defined L1-3 layers, *bzr-h* anthers displayed an L1 layer with randomly distributed cells and lacked differentiated L2 and L3 layers, resulting in the absence of archesporial cell specification (Chen et al. 2019). Unlike the anther phenotypes observed for *qui-1* and *bzr-h* mutant, the anther development of the BR receptor null mutant *bri1 brl1 brl3* (*bri-t*) was almost indistinguishable from the wild-type (WT) (Chen et al. 2019). Given that BRI1, BRL1 and BRL3 are the only known BL receptors, it was proposed that BZR TFs function in anther formation independent these receptors (Chen et al. 2019; Chen et al. 2019). Moreover, qRT-PCR analysis revealed that *SPL* expression was significantly reduced in *bri-t* unopened flower buds and almost undetectable in *bzr-h*, suggesting that *SPL* is regulated via BR signaling–dependent and BR signaling–independent pathways through BZRs (**Fig. 1A**) (Chen et al. 2019).

The BZRs were also shown to regulate tapetal development in the same pathway as the module TAPETUM DETERMINANT 1 (TPD1)-EMS1-SERK1/2 (Chen et al. 2019). Similar to BZR hextuple mutants, loss of EMS1, its cysteine-rich peptide ligand TPD1 and SERK1/2 resulted in anthers that are devoid of the tapetum layer (Canales et al. 2002, Zhao et al. 2002, Albrecht et al. 2005, Colcombet et al. 2005, Sun et al. 2010, Huang et al. 2016, Li et al. 2017). Consistent with BZR function downstream of the TPD1-EMS1-SRK1/2 module, introduction of the *bes1-D* and *bzr1-D* mutations in the *tpd1, ems1* and *serk1/2* backgrounds not only partially recovered a functional tapetum in these mutants but also reconstituted the expression of tapetal marker genes that were strongly downregulated in the single mutants (Chen et al. 2019). This was further supported by the observation that transgenic *bri1-116* lines expressing TPD1 and EMS1 under the *BRI1* promoter accumulated dephosphorylated BES1, thus mimicking a BR-dependent response without any exogenous BL application. Therefore, BES1 could be activated by the TPD1-EMS1-SERK1/2 module, independent of BR signaling (**Fig. 1A**) (Zheng et al. 2019, Chen et al. 2019).

### Roles of BR in the formation of the gynoecium and ovule primordia

Ovule primordia arise from the placenta as lateral organs (**Fig. 1B**) (Cucinotta et al. 2014), and the placenta itself originates from a meristematic tissue on the lateral margins of the carpel. This tissue, also known as the carpel margin meristem, additionally gives rise to the septum and to the transmitting tract (Cucinotta et al. 2014). The formation of the placenta is followed by the establishment of the regions where the ovule primordia will originate, which are interspersed by regions devoid of ovules (Cucinotta et al. 2014). These two developmental steps determine the total number of ovules per pistil and, upon fertilization, the number of seeds per silique. A role for BRs in regulating the ovule number and seed set has been demonstrated in several studies. While ovule/seed number is decreased in mutants with deficient BR biosynthesis and signaling, namely, in *det2, bri1-5* and *bin2-1*, the opposite is observed in the constitutive BR signaling mutant, *bzr1-D* (**Fig. 1B**) (Huang et al. 2013). In agreement with a positive role for BRs in this process, in *bzr1-D* suppressor lines, the number of ovules was reduced comparatively to the single mutant (Huang et al. 2013). Furthermore, pistils and siliques of transgenic lines with increased BZR1 dephosphorylation bore more ovules and seeds, and chemical inhibition of BIN2 activity in *bin2-1* resulted in a similar phenotype (Huang et al. 2013). Similarly, *Arabidopsis* plants with enhanced BR signaling, due to the expression of the *Glycine max* (soybean) *AtBZR1-LIKE GENE 2* (*GmBZL2*) harboring a point mutation in the conserved proline residue (thereby mimicking the *Arabidopsis bzr1-D* mutation), also showed an increased seed number per silique (Zhang et al. 2016). Even though ovule number was not directly quantified, the increase in seed number per silique is most likely caused by the presence of more ovules. Interestingly, overexpression of the *Zea mays* (maize) *DWF4* gene in *Arabidopsis* also enhanced seed yield, suggesting a conserved function for BR in regulating ovule/seed number in different species (Liu et al. 2007). However, in this case, it is not clear if each silique bore more seeds or if this increase was simply due to an increased number of siliques per plant. An increase in the seed set was also reported for *Arabidopsis* transgenic lines overexpressing *BR6OX2* (Kim et al. 2005).

The role of BR in determining the ovule number seems to be evolutionarily conserved as BRs also stimulate the seed set in crops like soybean and maize (Liu et al. 2007, Zhang et al. 2016) and promote ovule initiation in *Solanum lycopersicum* (tomato) (Barro-Trastoy et al. 2020a). The tomato cultivar Micro-Tom (MT) carries a mutation in the *DWARF4* gene leading to a reduced ovule number when compared to MT lines with the introgressed WT *DWARF4* (MT-D). Exogenous application of BL led to an increase in the ovule number in both lines, but this was more prominent in the MT background, supporting the notion that low levels of BRs reduce ovule primordia initiation and that this decrease can be compensated by BL treatment (Barro-Trastoy et al. 2020a). The BR-mediated regulation of ovule number in tomato was shown to occur through the downregulation of gibberellin (GA) levels: not only the endogenous GA levels were reduced in MT-D comparative to MT, but GUS activity was also decreased upon BR treatment in transgenic lines harboring a translational reporter for *SlGA20ox1*, a key GA biosynthesis gene (Barro-Trastoy et al. 2020a). However, since the *SlGA20ox1* promoter does not contain the BR *cis*-response elements bound by BZRs, the mechanism by which BRs repress *SlGA20ox1* transcription remains elusive. Contrarily to tomato, in *Arabidopsis*, the function of BRs in ovule primordia initiation is independent of GA (Barro-Trastoy et al. 2020b). Consistent with BRs promoting ovule primordial growth, the expression of *AINTEGUMENTA* (*ANT*) and *HUELLENLOS* (*HLL*), two genes involved in this developmental process, was induced in *bzr1-D* inflorescences (Huang et al. 2013). The expression of these genes was also enhanced in WT inflorescences treated with BL. Unlike for *HLL*, ChIP experiments revealed that *ANT* was a direct target of BZR1, hinting that BRs could regulate ovule number via this TF (Huang et al. 2013). However, Barro-Trastoy, et al. (2020a) demonstrated that this is likely not the case, since the ectopic expression of *ANT* did not alter the ovule number. Even though *ANT* activity seems to be required for promoting ovule primordia, it is likely by ensuring proper development of the placenta (Barro-Trastoy et al. 2020a).

It has also been recently proposed that BRs regulate ovule and seed number partially through a cytokinin (CK)-mediated pathway. Similar to the *bzr1-D* phenotype, in the *ckx3 ckx5* double mutant, in which CK levels are increased due to loss of CK-degrading oxidases/dehydrogenases (Bartrina et al. 2011), the siliques are longer than in the WT and have more seeds and ovules (Zu et al. 2022). Strikingly, the ovule/seed number per silique was further enhanced in the *bzr1-D ckx3 ckx5* triple mutant, despite the length of the siliques being indistinguishable from the *ckx3 ckx5* ones (Zu et al. 2022): in triple mutant siliques, the space in between ovules was reduced, likely creating space for more ovules, and the outermost layer of the pistil had an increased cell number compared to the *bzr1-D* or *ckx3 ckx5* mutants. Therefore, BRs and CKs promote ovule initiation and placenta elongation on their own, but that effect is synergistic (Zu et al. 2022). This is further supported by evidence that ARABIDOPSIS RESPONSE REGULATOR 1 (ARR1), a CK-response TF and interactor of BZR1, accumulates when CK treatment is combined with BR application (Zu et al. 2022). Conversely, an accumulation of phosphorylated BZR1 was also observed in *ckx3 ckx5* mutants, reinforcing that the two hormones act in a synergistic manner (Zu et al. 2022). Transcriptomic analysis revealed that genes involved in ovule initiation and identity, such as *HLL* and *SHATTERPROOF 2* (*SHP2*), which are neither differentially expressed in *bzr1-D* nor *ckx3 ckx5*, were upregulated in the triple mutant (**Fig. 1B**) (Zu et al. 2022). These observations indicate that the combinatory effect of both hormones induces the expression of genes that are not induced by either of the hormones individually. Nevertheless, the *ckx3 ckx5* mutant could partially complement the reduced seed phenotype of *det2* and *bin2-1*, but the ectopic CK degradation in *bzr1-D* did not alter seed number per silique, indicating that BRs regulate ovule/seed number via both CK-dependent and CK-independent pathways (Zu et al. 2022).

The transmitting tract, together with the stigma, style and funiculus, constitute the gynoecium reproductive tract, which is essential for proper fertilization (**Fig. 1B**). Among the genes required for the development of the reproductive tract is the one encoding the bHLH TF HALF FILLED (HAF) (Crawford and Yanofsky 2011). The siliques of *haf* mutants are smaller than WT ones and only contain seeds in the upper part of the silique (Crawford and Yanofsky 2011). Curiously, this female-specific phenotype was only observed in the Landsberg ecotype, since introgressing the *haf* allele into Columbia resulted in fully fertile siliques (Crawford and Yanofsky 2011). This observation indicates that, in contrast to the Landsberg ecotype, in Columbia, there are other genes acting redundantly with *HAF*. It was proposed that the BR-responsive bHLH TFs BRASSINOSTEROID-ENHANCED EXPRESSION1 (BEE1) and BEE3 could fulfill that function (Crawford and Yanofsky 2011). Even though fertility is not affected in the *bee1 bee3* double mutant, in the *haf bee1 bee3* (*hbb*) triple mutant, the number of seeds was strongly reduced and, just like in the Landsberg *haf* mutant, they localized to the upper region of the silique (Friedrichsen et al. 2002, Crawford and Yanofsky 2011). In agreement with a redundant function of these TFs, their expression patterns in the reproductive tract were shown to overlap: *HAF* expression, which occurs in all reproductive tract tissues, overlaps with that of *BEE1* in the stigma and style and with *BEE3* in the style and transmitting tract (Crawford and Yanofsky 2011). Morphological analysis of *hbb* transmitting tracts revealed that the cells fail to normally produce the extracellular matrix and to undergo programmed cell death, both of which are necessary for pollen tube growth (Crawford et al. 2007, Crawford and Yanofsky 2011). In agreement with these defects, pollen tube germination and growth through the style were reduced, as well as the apical–basal growth along the transmitting tract. Pollen tube elongation was constrained to a narrow path at the center of the transmitting tract, which was further reduced with the increasing distance from the style (Crawford and Yanofsky 2011). Despite the existing evidence, the mechanism by which HAF, BEE1 and BEE3 function in reproductive tract development is still unclear. Even though BEE1 and BEE3 are known to be induced by BRs, they are also activated by other hormones, suggesting that these TFs function in other hormonal pathways (Friedrichsen et al. 2002). Moreover, unlike *BEE1* and *BEE3, HAF* was not activated by BR application in seedlings (Friedrichsen et al. 2002). Even though this effect could be tissue-dependent, if HAF and BEEs were to interact and act together in reproductive tract development via BRs, one could expect that BL application would activate the BEEs, which could in turn activate HAF. As so, it is more likely that HAF and BEEs share target genes in reproductive tract development rather than jointly regulating gene expression. Nevertheless, it is still unclear if the function of the BEEs in the reproductive tract is in fact linked to BR responses.

### BRs contribute to the specification of the maternal germline

The ovule primordium can be described as a finger-like protrusion originating from the sporophytic placental tissue, where three distinct regions are established: the funiculus, which attaches the ovules to the placenta and establishes communication between the ovule and the maternal plant; the chalaza, a middle region from where the integuments originate; and the nucellus, which contains the megaspore mother cell (MMC) at the apex (**Fig. 1B**) (Schneitz et al. 1995). The MMC defines the female germline, and its differentiation initiates with the recognition and expansion of a germline cell precursor, followed by its commitment and transition into gametophytic development (Pinto et al. 2019). Therefore, the MMC can be distinguished from the surrounding somatic cells due to its larger size and dense cytoplasm (Drews and Koltunow 2011). Additionally, cells surrounding the MMC must be restricted from acquiring the germline fate, ensuring the specification of a single cell (Pinto et al. 2019). BRs were shown to repress germline fate in cells surrounding the MMC, thereby participating in the last event leading to MMC specification (**Fig. 1B**) (Cai et al. 2022). The ovule primordia of the BR biosynthesis mutants *det2-9, cpd, cyp85a2, dwf4* and BR signaling mutants *bri1-116* and *bzr1 bes1 beh1 beh3 beh4 (qui-1)* display multiple enlarged MMC-like cells that had the molecular characteristics of MMCs, as revealed by the expression of MMC marker genes (Cai et al. 2022). However, these MMC-like cells are unable to undergo meiosis, suggesting that they are not functional MMCs, but rather enlarged somatic cells that share some molecular properties with the MMC (Cai et al. 2022). Interestingly, the expression of genes encoding BR biosynthetic enzymes and components of the signaling pathway was restricted to the sporophytic tissues of the young ovules (Cai et al. 2022): while BR biosynthesis genes were detected in the epidermis and/or L1 layer of the nucellus region, the BR signaling genes were further observed in the subepidermal (L2) layers surrounding the MMC, suggesting that BRs repress L2 cells from acquiring germline fate. In addition, ectopic activation of BR signaling in the MMC resulted in ovule primordia with supernumerary MMC-like cells, via the BZR1-mediated activation of the germline cell fate repressor WRKY23 (Cai et al. 2022). Remarkably, the MMC-like cells of the *qui-1* mutant not only proceeded to meiosis but also initiated megagametogenesis (Cai et al. 2022). Even though the mature ovules lacked an embryo sac, likely due to its collapse or degeneration in later stages of gametophyte development, these observations point to additional functions of the BZR family members in entry to meiosis and in gametogenesis.

Recently, a link between BRs and the ERECTA family (ERf) signaling pathways in MMC specification has also been demonstrated (Cai et al. 2023). Similar to previous findings, ovule primordia of high-order mutants for the receptors ER, ERECTA-LIKE1 (ERL1) and ERL2 and their ligands EPIDERMAL PATTERNING FACTOR (EPF)/EPF-LIKE (EPFL) displayed multiple MMC-like cells that shared molecular properties with the MMC (Cai et al. 2023). Interestingly, *BRI1* and *BZR1* expression was greatly reduced in *er erl1 erl2* and *epfl1,2,4,6* mutants, while *ER* expression in *bri1-116* was indistinguishable from the WT, indicating that BR signaling acts downstream of ERf (Cai et al. 2023). Moreover, *bzr1-D* and *bes1-D* mutations, which lead to increased BR signaling (Wang et al. 2002, Yin et al. 2002), partially restored the multiple MMC-like phenotype observed in *er erl1 erl2* and *epfl1,2,4,6* mutants, reinforcing the notion that BR signaling acts downstream of ERf (Wang et al. 2002, Yin et al. 2002, Cai et al. 2023). In agreement with BR and ERf jointly regulating MMC specification, concomitant loss of *ER* and *BRI1* increased the frequency of MMC-like cells in comparison to the WT and to the single mutants (Cai et al. 2023). This phenotype could once again partially be restored by *bzr1-D* and *bes1-D* mutations (Cai et al. 2023). It was further revealed that BZR1 and BES1 target the promoter of *NUCLEOSTEMIN-LIKE1* (*NSN1*), which encodes a nuclear GTP-binding protein, whose role is to restrict MMC surrounding cells from acquiring germline fate (**Fig. 1B**) (Zhao et al. 2021, Cai et al. 2023). Although it is becoming increasingly clear how BRs contribute to the specification of the maternal germline, the molecular mechanisms of how the ERf pathway activates BR signaling remain to be elucidated.

### Regulation of ovule development by BR

The differentiation of the MMC is followed by its entry to meiosis, giving rise to four megaspores from which only one survives. The functional megaspore undergoes three rounds of mitotic divisions to originate the eight nuclei that constitute the FG (Christensen et al. 1997). The first round of mitosis results in two daughter nuclei that are separated by a large central vacuole, which originates from the gradual fusion of several smaller vacuoles in the center of the cell (Christensen et al. 1997). The formation and expansion of the central vacuole are crucial for FG development as it influences the expansion of the FG and the positioning of the different nuclei within the embryo sac (Hu et al. 2023). The GSK3-like kinase BIN2 and its close homologs BIN2-LIKE1 (BIL1) and BIL2 were shown to regulate the formation of the central vacuole through the stabilization of VACUOLELESS GAMETOPHYTES (VLG), a protein that promotes vesicular fusion in multivesicular bodies or pre-vacuolar compartments to form the large FG vacuole (Jonak and Hirt 2002, D’Ippólito et al. 2017, Hu et al. 2023). In contrast to WT FGs, in which the large vacuole is mostly observed after the first nuclear division, in *bin2-1*, a dominant mutation leading to constitutively active BIN2 (Li et al. 2001), a vacuole was already observed prior to division, indicating that ectopic activation of BIN2 results in precocious vacuole formation (Li and Nam 2002, Hu et al. 2023). This notion was further supported by the observation that expressing the *bin2-1* allele in WT FGs induced the formation of aberrant vacuoles, a phenotype whose frequency could be reduced upon treatment with the GSK3 kinase inhibitor, bikinin (Hu et al. 2023). BIN2 was shown to interact and phosphorylate VLG, thereby protecting it from proteasomal degradation (Hu et al. 2023). The increased stability of phosphorylated VLG was further demonstrated when a phosphomimetic version of VLG was expressed in WT FGs, resulting in defects similar to those observed in *bin2-1* (Hu et al. 2023). Interestingly, both gain and loss of BIN2 lead to FG degeneration phenotypes: in the *bin2-1* gain-of-function mutant, VLG stability is increased, reducing its proteasomal degradation and leading to precocious large vacuole formation; in contrast, in the loss-of-function *bin2-3 bil1 bil2* mutant, VLG is ectopically degraded resulting in arrested vacuole formation (Hu et al. 2023).

The embryo sac develops in coordination with the maternal integuments (Schneitz et al. 1995). *Arabidopsis* ovules are bitegmic and thus produce two integuments: the inner one initiates development first and is shortly afterward followed by the outer integument (**Fig. 1B**) (Schneitz et al. 1995). In contrast to the inner integuments, which grow equally in all directions, giving rise to symmetric layers around the nucellus, the growth of the outer integument is more pronounced in the abaxial side (Schneitz et al. 1995). This differential growth of the outer integument leads to a curved mature ovule, with the micropyle placed close to the funiculus (**Fig. 1B**) (Schneitz et al. 1995, Vijayan et al. 2021). The ovules of *bri1-116, bri1-5, bzr-h, det2, dwf4-44* and *br6ox2* mutants display defective outer integument growth, linking BR to the development of this tissue (Nole-Wilson et al. 2010, Jia et al. 2020, Lima et al. 2023). One of the most striking phenotypes is observed in *bri1-116* ovules, in which a wide range of phenotypes could be distinguished, ranging from ovules with mild to severe outer integument growth arrest (Jia et al. 2020). The most severe phenotype included ovules with more than half of the inner integument surface exposed (Jia et al. 2020). In addition, *bri1-116* ovules often display abnormal embryo sacs (Jia et al. 2020). This can potentially be attributed to the formation of supernumerary MMC-like cells recently described by Cai et al. (2022), but it is also well established that integument malformations can lead to female sterility (Robinson-Beers et al. 1992). Even though WT-like ovules could also be found in *bri1-116* ovaries, they still showed a reduced curvature and some were unable to attract pollen tubes (Jia et al. 2020). The defective outer integument phenotype was caused by the reduced cell number and length, indicating that in outer integument growth, BRs likely affect both cell division and elongation (Jia et al. 2020). A similar, yet milder, phenotype was described for *br6ox2* mutants: albeit most ovules resembled the ones in WT, a small percentage showed slightly reduced outer integument growth and lacked embryo sacs (Nole-Wilson et al. 2010). Whether the shorter outer integument is also caused by reduced cell elongation and/or division remains to be determined. Constitutive BR signaling through *bzr1-D* could partially alleviate the strong ovule phenotypes seen in *bri1-116*, revealing that BZR1 functions downstream of BRI1 in outer integument and embryo sac development (Jia et al. 2020). Remarkably, loss of *BRI1, BRL1* and *BRL3* resulted in outer integument defects identical to the ones observed for *bri1-116* ovules, but these defects were further enhanced in the *bzr-h* mutant, suggesting that just like in loculi formation, a BRI1-independent pathway might regulate BZR activity in the development of the maternal sporophytic tissues (Chen et al. 2019, Jia et al. 2020). Interestingly, *BRI1* and *BZR1* expression was detected in both the inner and outer integuments (Jia et al. 2020, Lima et al. 2023), despite only outer integument growth being affected in *bri1-116* and *bzr-h* mutants, indicating that BRs mostly regulate the expression of outer integument specific genes (Jia et al. 2020). Indeed, the expression of *INNER NO OUTER* (*INO*), a crucial gene in outer integument development (Villanueva et al. 1999) and direct target of BZR1, was almost undetectable in *bzr-h* mutants, suggesting that BRs regulate outer integument growth by modulating *INO* expression via the BZRs (**Fig. 1B**) (Jia et al. 2020). In agreement with this hypothesis, the overexpression of INO strongly reduced the frequency of *bri1-116* ovules showing severe outer integument growth (Jia et al. 2020). Notably, the severity of the outer integument phenotype did not correlate with the embryo sac defects, revealing that BR-mediated regulation of outer integument and gametophyte development likely occurs via distinct BZR targets (Jia et al. 2020).

Interestingly, defective embryo sacs have also been reported in the gain-of-function mutant *bzr1-D*. In *bzr1-D* ovaries, ovules with a persistent nucellus or two nuclei instead of a single central cell were often found (Lima et al. 2023). These phenotypes are consistent with underdeveloped ovules, suggesting that excessive BR signaling is also detrimental for embryo sac formation. Given that *bzr1-D* could partially complement the defective gametophytes of *bri1-116* ovules (Jia et al. 2020), it is likely that BR-mediated regulation of embryo sac development occurs in a dose-dependent manner.

The fact that the FG is protected by many layers of cells creates a difficult environment to study its development. Nevertheless, in recent years, more detailed studies aiming to understand the function of BR in ovule development have started to emerge. It is noticeable that in most studies, the expression of the BR signaling components has been restricted to the sporophytic tissues of the ovule, hinting that the effect of BRs in embryo sac development is likely non-cell autonomous. Moreover, the overall evidence suggests that integument growth and gametophyte development are likely regulated by different BZR target genes. Therefore, more detailed analysis of BR mutant ovules such as from *bzr1-D*, which have defective gametophytes yet WT-looking integuments (Lima et al. 2023), could be helpful in uncoupling mechanisms by which BRs regulate integument and gametophyte development.

## BR Regulates Maternal–Paternal Interactions

### Pollen tube formation is modulated by BRs

As alluded earlier, studies of BR biosynthesis, perception and signaling mutants have been indicative of male infertility (Szekeres et al. 1996, Bouquin et al. 2001, Li et al. 2001, Ye et al. 2010). While this is in part due to development defects during anther and pollen development, as discussed earlier, loss of BR function has long been known to affect pollen germination and pollen tube formation (Szekeres et al. 1996). Indeed, exogenous application of BRs influences the germination rate of pollen grains and the size of their tubes in a variety of species, including several crops like cherry (Hewitt et al. 1985), tomato (Singh and Shono 2005), grapevine (Gokbayrak and Engin 2016), mango (Tepkaew et al. 2022) and rice (Thussagunpanit et al. 2013), as well as in *Primula* (Huu et al. 2022). The same is true in the model species *Arabidopsis*, where the effect of exogenous BRs on both the pollen germination and tube growth rates seems to be dose-dependent, with too low or too high doses being inhibitory (Vogler et al. 2014). Importantly, the regulation of pollen germination and tube growth by BRs may be due to non-cell autonomous effects from the pistils. As we discussed in the section on ovule development, lack of BRs leads to defects in the maternal reproductive tract. Consistent with this, *CPD* and *BRI1* are more strongly expressed in *Arabidopsis* pistils than in pollen (Vogler et al. 2014). Indeed, *CPD* is strongly expressed both in the stigmatic papillae, where the pollen germinates, and in the conducting tracts, through which the pollen tubes grow (Vogler et al. 2014). Moreover, the growth of WT pollen tubes is significantly impaired when germinated in *cyp90a1-1* (*cpd*) or *bri1-10* mutant pistils (Vogler et al. 2014), but *cpd* or *bri1-116* pollen tubes grow normally in WT pistils (Ye et al. 2010). These observations link the pollen phenotypes to maternal BR production and signaling. Since long, it has been observed that the germination and growth of pollen tubes in vitro are greatly enhanced by the addition of exudates of maternal pistil tissues (Tsukamoto and Matsubara 1968, Martin and Brewbaker 1971). This is the reason why semi-in vivo systems are necessary for pollen germination and targeting assays, where the pollen is germinated on a cut pistil, through which the pollen tubes grow (Higashiyama et al. 1998, Palanivelu and Preuss 2006). It is thus reasonable to hypothesize that BRs are one of the maternal signals that are necessary for efficient pollen germination and for the growth and targeting of the pollen tubes. This has further implications, as we discuss in the section on reproductive barriers, as BR-mediated maternal regulation of pollen tube formation thus underlies hybridization barriers in several species. However, it is of note that the molecular mechanisms by which BRs create a permissive environment for pollen tube formation have not been fully elucidated.

### Maternal BRs underlie reproductive barriers

Self-incompatibility (SI) systems can be shaped by BR activity, and, as alluded earlier, this seems to be intrinsically linked to the regulation of pollen germination and pollen tube growth by BRs. SI can be broadly divided into homomorphic and heteromorphic systems (Lewis 1949). In the former, there are no differences in flower morphology, while in the latter, the incompatible types are normally indicated by different morphologies (or morphs) (Barrett 2002). One such example is heterostyly, where one morph has short anthers and long styles (l-morph) and the other morph has the opposite arrangement of the flower organs (s-morph) (**Fig. 2A**). Efficient fertilization is only possible between different morphs. This type of SI is widespread in several plant families and is particularly well understood in some species of primrose (*Primula*) (Kappel et al. 2017, Barrett 2019). Heterostyly in *Primula* is determined by the presence or absence of a hemizygous *S*-locus supergene, which, among other traits, controls stamen and style length (Nowak et al. 2015). Among the genes in the *S*-locus is *CYP734A50*, which encodes a cytochrome P450, implicated in degrading BRs (Ohnishi et al. 2006a, Thornton et al. 2011). Importantly, the *S*-locus is characteristic of s-morphs, meaning that *CYP734A50* is only expressed in those individuals, and this correlates with reduced endogenous levels of CS (Huu et al. 2016). Consistently, the short style phenotype can be rescued by the exogenous application of epi-BL and the loss of function of *CYP734A50* resulted in lengthening of the styles (Huu et al. 2016). In *Arabidopsis*, the loss of function of the BR biosynthesis gene *BR6OX2* leads to different growth rates of the stamens compared to the style (Kim et al. 2005). Although in this case, lower BR levels in the *Arabidopsis cyp85a2* mutants lead to a phenocopy, an l-morph, rather than of an s-morph, this is an indication that the modulation of BR levels can be an efficient way to shape flower morphology and could potentially underlie heteromorphism in other SI systems.

Strikingly, BR function not only determines the morphology of the *Primula* flowers but also the incompatibility type: short-styled individuals losing *CYP734A50* function not only form long styles but also acquire the female compatibility of the long-styled individuals (Huu et al. 2022). The precise molecular mechanisms by which BRs control SI are still not fully understood, but they are likely linked to an indirect effect of BRs on pollen germination and to a direct effect on pollen tube growth (Huu et al. 2022). Because heterostyly only evolved once in *Primula* (Mast et al. 2006), it is likely that the SI system in this genus is tightly linked to BR function. In another heterostylous species, *Turnera*, style length and female incompatibility were also linked to BR activity (Matzke et al. 2020, 2021). However, in this case, the determinant seems to be a BAHD acetyltransferase, also linked to inactivation of bioactive BRs, both in *Arabidopsis* and in *Turnera* (Roh et al. 2012, Matzke et al. 2020). This suggests that the modulation of BR levels may be a relatively common pathway for the evolution of heteromorphic SI.

Interestingly, BRs have also been implicated in a different type of SI, namely, in S-RNase-mediated SI (Wang et al. 2023). S-RNases are style-specific ribonuclease proteins that are transported to the germinating pollen tube, where they are processed differently according to compatibility (Kao and Tsukamoto 2004, Meng et al. 2014). While nonself or compatible S-RNAses are targeted for proteasomal degradation, the self or incompatible ones cause pollen tube growth arrest due to their cytotoxic properties (Gray et al. 1991, Lai et al. 2002; Liu et al. 2014, Sijacic et al. 2004). In pear (*Pyrus*), exogenous application of epi-BL suppressed the inhibitory effects of SI on pollen tube growth, and this effect is impaired if the BR effector PbrBZR1 is knocked-down (Wang et al. 2023). Pollen tube growth in pear is thus thought to be driven by the activation of expansins by PbrBZR1 (Wang et al. 2023). While the exact mechanisms by which pollen tube growth and germination are controlled are still unknown, it is tempting to hypothesize that modulation of BR homeostasis could be a simple one-step mechanism to control pollen tube formation and could therefore underlie hybridization barriers in a wide range of species.

## Regulation of Seed Development by BRs

There is considerable phenotypic evidence that BRs regulate seed formation: mutants affected in BR homeostasis often show changes in seed size and shape. This has been seen in several species, namely, in rice (Hong et al. 2005, Tanabe et al. 2005, Wu et al. 2008, Zhang et al. 2018, 2022), pea (Nomura et al. 2007), faba bean (Fukuta et al. 2006) and *Arabidopsis* (Choe et al. 2000, Takahashi et al. 2005, Jiang et al. 2013, Pankaj et al. 2023). Consistent with this, the level of bioactive BRs gradually increases during seed development in pea (Nomura et al. 2007). However, the underlying molecular mechanisms by which BRs control seed growth remain relatively unexplored, compared to what is known about how these hormones shape the development of other organs. Angiosperm seeds are composed of three structures: embryo, endosperm and seed coat (**Fig. 2B**). In this section, we focus on the regulation of endosperm and seed coat development by BRs. This is because, surprisingly, although these hormones have been implicated in several aspects of plant development, there is limited information on their regulation of zygotic embryogenesis. Embryos originating from the seeds of BR mutants are often smaller than their WT counterparts, but this is likely an indirect effect of BRs on seed coat expansion, which creates a smaller cavity for the embryo to grow in. Indeed, as we discuss later, the modulation of seed size by BRs is mostly likely a result of a sporophytic effect of the seed coat, although zygotic effects have also been proposed in some instances.

In *Arabidopsis*, lack of BR biosynthesis and signaling has been shown to affect both seed coat expansion and its shape (Choe et al. 2000, Takahashi et al. 2005, Jiang et al. 2013, Pankaj et al. 2023). This results in BR mutant seeds being smaller and rounder, as opposed to their elongated characteristic WT phenotype (Choe et al. 2000, Jiang et al. 2013, Pankaj et al. 2023). Similarly, ectopic expression of *SHRINK1* (*SHK1*), encoding a cytochrome P450 monooxygenase homolog to the BR degrading enzyme PHYB-4 ACTIVATION-TAGGED SUPPRESSOR 1 (BAS1), also leads to smaller and rounder seeds (Takahashi et al. 2005, Turk et al. 2005). Consistently, plants expressing the *shk1-D* dominant allele have lower endogenous levels of BRs (Takahashi et al. 2005). Importantly, BR mutant mature ovules are of the same size as their WT counterparts, meaning that the reason why the seeds are smaller has to do with effects on post-fertilization growth and not on the initial size of the ovules (Pankaj et al. 2023). Interestingly, the seed shape phenotype of the *det2* and *bri1-116* BR mutants was initially proposed to be maternally controlled, while their size would be under zygotic control (by the endosperm or the embryo) (Jiang et al. 2013). In fact, genes encoding endosperm-specific seed size regulators were proposed to be the targets of BZR1, linking BR signaling to seed expansion (Sun et al. 2010, Jiang et al. 2013). This included genes encoding the components of the endosperm-specific HAIKU (IKU) pathway, like *IKU1, IKU2* and *SHORT HYPOCOTYL UNDER BLUE 1* (Garcia et al. 2003, Zhou et al. 2009), as well as those encoding the growth regulators AP2 and ARF2 (Ohto et al. 2005, Schruff et al. 2006). However, both BR biosynthetic and signaling machinery seem to be specifically expressed in the seed coat during early stages of seed development and not in the endosperm or the embryo (Lima et al. 2023). Moreover, these early seed size defects of BR mutants can be rescued by seed coat–specific complementation of *det2-1* and *bri1-6* (Pankaj et al. 2023). It is therefore possible that seed size is determined by BR signaling in the sporophytic tissues during early seed development, but by zygotic effects in later stages. It remains to be tested in which seed tissues are the BR biosynthesis and signaling genes expressed at later stages of development.

Regarding the early regulation of seed size by BR, namely, as determined by the sporophytic seed coat, it seems to be linked to the removal of the repressive epigenetic mark, trimethylation of lysine 27 on histone 3 (H3K27me3). This mark is deposited by the Polycomb Repressive Complex 2 (PRC2) in the ovule integuments, which suppresses seed coat formation in the absence of fertilization (Roszak and Köhler 2011, Figueiredo et al. 2016). Consistent with this, loss of PRC2 function leads to fertilization-independent seed coat formation, and to its increased growth, leading to larger seed sizes (Roszak and Köhler 2011, Figueiredo et al. 2016, Pankaj et al. 2023). Recent observations propose that the H3K27me3 marks must be actively removed after fertilization, in order for the seed coat to grow (Pankaj et al. 2023). Enzymatic removal of such marks is carried out by JUMONJI histone demethylases (JMJ), which have been shown to genetically and physically interact with the BR effectors BZR1 and BES1 (Yu et al. 2008, Li et al. 2018). More specifically, BZR1 interacts with the JMJ protein EARLY FLOWERING 6 (ELF6) (Li et al. 2018), while BES1 interacts both with ELF6 and with RELATIVE OF EARLY FLOWERING 6 (REF6) (Yu et al. 2008). Consistent with this, *elf6 ref6* mutants show seed coat growth defects, similar to BR mutants (Jiang et al. 2013, Pankaj et al. 2023). Importantly, the seed growth defects of *bri1-6* can be fully rescued by loss of sporophytic PRC2s, suggesting that those defects are indeed due to poor removal of H3K27me3 from the ovule integuments in the absence of BR signaling (Pankaj et al. 2023). Surprisingly, the same was not observed for the *det2-1* seed size phenotypes, indicating that the loss of PRC2 is epistatic to the loss of BR signaling via BRI1, but not to the loss of BR biosynthesis (Pankaj et al. 2023). This suggests that BRs control seed coat growth via both H3K27me3-dependent and H3K27me3-independent pathways, potentially controlled by different BR receptors (**Fig. 2B**). Indeed, another BR receptor, BRL3, is also expressed in the *Arabidopsis* seed coat, in addition to BRI1 (Lima et al. 2023, Pankaj et al. 2023). Consistent with a putative H3K27me3-independent regulation of seed growth, the *brl3* mutant also forms smaller seed coats, but this phenotype is not rescued by loss of PRC2, unlike what happens for *bri1-6* (Pankaj et al. 2023). Thus, both BRI1 and BRL3 control seed growth, by modulating seed coat expansion, but this likely occurs via independent molecular mechanisms (**Fig. 2B**).

It is important to note that higher-order *jmj* mutants are characterized by dwarf phenotypes, similar to those observed in BR mutants (Yan et al. 2018, Pankaj et al. 2023). The dwarf phenotypes of BR mutants are alleviated by the loss of PRC2 function and thus loss of H3K27me3 (Pankaj et al. 2023). This suggests that multiple BR mutant phenotypes, namely, those of *bri1* mutants, may be related to the hypermethylation of H3K27. Together with mounting evidence that BRI1-LIKE receptors carry out specific functions during plant development (Fàbregas et al. 2013, 2018), it is very likely that BR regulates development via H3K27me3 homeostasis, but that other pathways, e.g. via BRL3, shape BR signaling via mechanisms independent of those epigenetic marks.

In addition to regulating seed coat growth, BR has also been shown to control early endosperm proliferation, as mutants impaired in BR biosynthesis and signaling, *det2-1* and *bri1-6*, show slower endosperm nuclei replication than the WT (Lima et al. 2023). This fits with previous observations that genes involved in endosperm growth are downregulated in *det2* mutants, or after exogenous applications of the BR biosynthesis inhibitor brassinazole, and are upregulated upon exogenous epi-BL treatments (Jiang et al. 2013). Surprisingly, the effect of BRs on endosperm proliferation seems to be due to indirect effects of the seed coat, rather than the direct regulation of endosperm genes by BR effectors (Lima et al. 2023). Indeed, at those early stages of development, the receptor BRI1 and the effectors BZR1 and BES1 are only expressed in the seed coat; moreover, WT pollen cannot rescue the endosperm proliferation defects of BR mutants (Lima et al. 2023). Because the BR mutant seeds are smaller, due to the seed coat defects indicated earlier, the authors proposed that the physical size of the seed coat determines the endosperm proliferation rate. Consistent with this, BR mutant seed coats are depleted in methylesterified pectins, likely making them stiffer, and less prone to expansion (Lima et al. 2023). Observations in rice also suggest an indirect effect of BRs on grain filling (Wu et al. 2008). Ectopic expression of sterol C-22 hydroxylases, involved in BR biosynthesis, led to the production of larger grains (Wu et al. 2008). Importantly, this ectopic expression was done using the promoter of a *S-ADENOSYLMETHIONINE SYNTHASE*, which is not expressed in the endosperm or the embryo, but only in the vegetative tissues, including the seed coat (Wu et al. 2008). In fact, expression of the sterol C-22 hydrolases in the embryo or endosperm did not have an effect on seed filling (Wu et al. 2008). This indicates that BR-mediated sporophytic control of endosperm development may be a common feature in both monocots and eudicots. It is, however, possible that BRs directly regulate endosperm formation in other species. Although BZR1 was not found expressed in the endosperm of *Arabidopsis*, its homolog is expressed in tomato endosperm (Florez-Rueda et al. 2016). Moreover, a *BRL1* homolog is expressed in the endosperm of *Amborella trichopoda*, and homologs of *BRASSINOSTEROID*-*RELATED HOMEOBOX 2* (*BHB2*) are expressed in the endosperm of species of several clades (Florez-Rueda et al. 2024). The functional significance of BR signaling in the endosperm of other species has, however, not been assessed to date.

## BR Modulates Fruit Development and Ripening

In addition to their post-fertilization role in seed development, BRs have also been shown to regulate fruit formation, although the underlying molecular mechanisms remain relatively unexplored. Here, we focus on fleshy fruit formation in eudicots. Because in cereals, the pericarp is fused with the seed coat, we already indirectly addressed caryopsis-like fruit development in the seed development section. It should also be noted that although failure of silique elongation is a hallmark phenotype of BR mutants in *Arabidopsis*, this is often linked to other reproductive phenotypes, as we discussed in the ovule and seed development sections. Therefore, how much BRs contribute to silique formation in *Arabidopsis* has so far been poorly explored.

Overall, there is limited information on the regulation of early fruit development by BRs. This is likely in part due to other reproductive phenotypes, which mask any potential effects of a lack of BRs on fruit development. Interestingly, however, exogenous application of BRs was shown to induce parthenocarpy in cucumber, i.e. fruit development without pollination (Fu et al. 2008). Concomitantly, applications of Brassinazole to a parthenocarpic cultivar suppressed the asexual fruit phenotype (Fu et al. 2008). To our knowledge, this has not been reported elsewhere, potentially indicating that BRs could be a driving signal for fruit development in cucumber, but not in other species. However, a role for BRs in regulating fruit shape has been reported in tomato (Yu et al. 2022). The overexpression of a *BZR1* homolog, called *SlBZR1.7*, led to abnormal fruit elongation in tomato, and this was proposed to be due to the direct regulation by SlBZR1.7 of *SUN*, a regulator of fruit shape (Wu et al. 2011, Yu et al. 2022). Mutations in *SlBZR1.7*, however, did not result in obvious fruit shape phenotypes, suggesting functional redundancy with other SlBZRs (Yu et al. 2022). Supporting this, the overexpression of *SlBZR1.5* and *1.6* also leads to fruit elongation phenotypes, but the same did not happen for *SlBZR1.1, 1.2, 1.3* and *1.4* (Yu et al. 2022). Consistent with a role for BRs during early fruit development, BR biosynthesis genes are expressed in developing tomato fruits and these immature fruits accumulate high levels of BL (Montoya et al. 2005).

In addition to its role in determining the fruit shape in tomato, BRs have been implicated in fruit ripening in this species (Vidya Vardhini and Rao 2002, Liu et al. 2014, Zhu et al. 2015, Meng et al. 2023). For instance, exogenous application of epi-BL or the ectopic expression of the *Arabidopsis* dominant *BZR1-1D* was reported to alter the accumulation of carotenoids in tomato fruits (Liu et al. 2014, Zhu et al. 2015). Consistent with this, mutations in two tomato *BZRs*, termed *SlBZR1* and *SlBES1*, led to a reduction in carotenoid production and to a delay in fruit ripening, potentially via the regulation of the *Phytoene synthase 1* gene (Meng et al. 2023). Interestingly, while BR activity, likely via SlBZR1/SlBES1, seems to control tomato ripening via the promotion of ethylene biosynthesis, the control of carotenoid production is apparently done in an ethylene-independent manner (Zhu et al. 2015, Meng et al. 2023). Moreover, the effect of BRs on fruit ripening does not seem to be limited to tomato, as similar observations have been done in non-climacteric fruits like grapes and strawberries (Symons et al. 2006, Chai et al. 2013).

In addition to its role in regulating fruit ripening, BR signaling has also been implicated in fruit softening in tomato and in peach (Zhu et al. 2015, Liu et al. 2021, Li et al. 2023). In tomato, exogenous application of epi-BL induces fruit softening, while the application of brassinazole has the opposite effect (Zhu et al. 2015). Transgenic lines ectopically expressing *SlBES1* produce softer fruits, while those expressing RNAi constructs targeting *SlBES1* produced firmer fruits (Liu et al. 2021). The authors linked these phenotypes to modified pectin metabolism, as SlBES1 was shown to bind to the promoter of *PME ubiquitously 1*, which encodes an inhibitor of pectin methylesterase activity (Liu et al. 2021). Consistent with this, manipulation of pectin metabolism has been shown to regulate fruit softening in tomato (Uluisik et al. 2016, Wang et al. 2018). Interestingly, manipulation of different pectin degrading enzymes either affects fruit softening or fruit color, in the latter case due to differential accumulation of carotenoids (Wang et al. 2018). Therefore, it is likely that BRs regulate fruit ripening via the modulation of different pathways, with some determining carotenoid accumulation and others regulating fruit softening. Whether the BR regulation of all these processes is through the modulation of pectin metabolism remains to be tested. Indeed, proteomic analyses have implicated BR in regulating not only cell wall metabolism during fruit formation in tomato but also other hormonal pathways, as well as components of primary metabolism (Liu et al. 2016). Unexpectedly, exogenous application of epi-BL to peach fruits led to delayed softening (Li et al. 2023), which is the opposite of what is described for tomato (Zhu et al. 2015, Liu et al. 2021). Whether this means that BRs have opposing effects in fruit softening in different species, or if this was the result of a particular experimental set-up, remains to be studied further.

## BR as Potential Tools for Crop Improvement

In the previous sections, we discussed the current body of knowledge on how BRs shape reproductive development in plants. Importantly, the products of reproduction, fruits and seeds, are the basis of our diet, either directly or indirectly via animal fodder. This means that the optimization of reproductive outputs is of great socio-economic interest. In this section we discuss how manipulation of BRs can potentially have wide applications in several aspects of crop improvement.

### In bypassing hybridization barriers

Although phenotypes related to male fertility are characteristic of BR mutants, we are only starting to understand how BRs regulate pollen germination and growth. Importantly, BRs promote pollen germination in several crop species (Hewitt et al. 1985, Singh and Shono 2005, Thussagunpanit et al. 2013, Gokbayrak and Engin 2016, Tepkaew et al. 2022), meaning that BR manipulation could be a promising strategy to increase pollen viability. Moreover, BRs have been shown to be determinant for SI (Matzke et al. 2021, Huu et al. 2022). Bypassing hybridization barriers between certain species or cultivars is of great agricultural value (Muñoz-Sanz et al. 2020), and often breeders have to rely on naturally occurring mutations that break down SI systems. Therefore, it will be very exciting to see if tissue-specific manipulation of BR function can be a universal strategy to shape mating systems and thus allow breeders to widen the range of compatible crosses to generate new varieties.

### In increasing seed size and yield

One rather obvious trait where manipulation of BRs can lead to increased yields is in the control of seed size. As we discussed earlier, changes in BR activity can have direct effects on the size and shape of seeds. This has been shown both in eudicots (Noguchi et al. 2000, Takahashi et al. 2005, Fukuta et al. 2006, Nomura et al. 2007, Jiang et al. 2013, Lima et al. 2023) and in monocots (Hong et al. 2005, Tanabe et al. 2005, Wu et al. 2008, Zhang et al. 2018, 2022, 2024). Therefore, the manipulation of BR biosynthesis and signaling can be a promising tool to directly shape the size of seeds. In addition to this, because of the role of BRs in determining the ovule number in multiple species (Barro-Trastoy et al. 2020a; Kim et al. 2005, Liu et al. 2007, Zhang et al. 2016), engineering of seed number per fruit or panicle by manipulating BR activity is also a potential target trait for increasing yield. However, although manipulation of BR levels or signaling can have positive effects on seed size, this can come at the cost of seed number (Zhang et al. 2022). It will therefore be important to design strategies that allow for the manipulation of BR biosynthesis or signaling in a tissue-specific manner or at specific stages of development, to avoid unintended phenotypes. One example of such a strategy is the recently published manipulation of BR levels in rice via the BR catabolism gene *BRASSINOSTEROID-DEFICIENT DWARF4* (*BRD3*) (Zhang et al. 2024). The tissue-specific degradation of BRs in the secondary branch meristems, via the ectopic expression of *BRD3*, leads to an increase in grain number without compromising their size (Zhang et al. 2024). This elegant strategy showcases how tissue and temporal engineering of BR homeostasis has the potential to dramatically influence crop yield.

In addition to the direct effect of BRs in seed growth, manipulation of BR levels in vegetative tissues has also been shown to impact seed yield (Choe et al. 2001, Sakamoto et al. 2006, Wu et al. 2008, Zhang et al. 2024). For instance, constitutive overexpression of *DWF4* in *Arabidopsis* leads to increased branching and therefore more flowers and more seeds (Choe et al. 2001, Liu et al. 2007). Unexpectedly, the mutations *osdwarf4* or *d61* (an allele of *OsBRI1*) also increased grain yield in rice, in particular when plants were grown at high densities (Morinaka et al. 2006, Sakamoto et al. 2006). This was proposed to be due to the erect nature of *osdwf4* leaves, which allows for higher rates of photosynthesis when plants are densely packed (Morinaka et al. 2006, Sakamoto et al. 2006). This means that fine tuning of BR responses can be a viable strategy for optimizing yield in plants grown under different conditions. A recent study also showed that altered BR perception in wheat, due to the loss of a RING-type E3 ligase (encoded by *ZnF-B*), leads to compact plants without compromising grain yield (Song et al. 2023). Compact plant architecture is a hallmark of the Green Revolution and was achieved through the manipulation of gibberellin signaling (Peng et al. 1999). However, this phenotype comes at the cost of grain size. Song et al. (2023) demonstrated that loss of *ZnF-B* can be an alternative strategy to achieve a compact plant architecture, without the characteristic side effect of reduced seed size of gibberellin mutants, again demonstrating that the efficient manipulation of BR homeostasis is a promising avenue for manipulating yield in seed crops.

### In regulating parthenocarpy

BRs were also implicated in promoting fruit development without pollination, or parthenocarpy, in cucumber (Fu et al. 2008). This is a highly desirable trait in the Cucurbitaceae (Tian et al. 2023), as it leads to the development of seedless fruits, which is popular among consumers. Moreover, pollen production is highly sensitive to heat (Rieu et al. 2017), meaning that global warming can have devastating consequences in crops whose production relies on efficient pollination. The same applies to situations where pollinators are absent. Thus, the development of crops that can maintain fruit yield even under adverse environmental conditions will be of great value. However, it should be noted that the effect of BRs in driving parthenocarpy has not been demonstrated in species other than cucumber. Whether this effect of BRs is particular to that species, or to the Cucurbitaceae, is yet to be explored.

### In increasing shelf life

Manipulation of BR activity can also be a promising method to increase post-harvesting traits. As mentioned in the section on fruit development section, BRs control fruit ripening, in both climacteric and non-climacteric species (Chai et al. 2013; Liu et al. 2014, Meng et al. 2023, Symons et al. 2006, Vidya Vardhini and Rao 2002, Zhu et al. 2015). Controlling fruit ripening is highly desirable because it determines the shelf life of many products (Adaskaveg and Blanco-Ulate 2023). In particular, because of the role of BRs in controlling fruit softening in tomato (Uluisik et al. 2016, Liu et al. 2021), the manipulation of this hormone can be a promising tool to increase shelf life in important food crops and therefore contribute to alleviating food waste.

As detailed throughout this review, BRs are important regulators of almost all aspects of plant reproduction. However, compared to the extensive body of knowledge on how BRs regulate aspects of vegetative development, relatively little is known about how these hormones shape reproduction. In particular, the molecular mechanisms underlying BR responses in reproductive structures remain vastly unexplored. Nevertheless, as we outline in this last section, the manipulation of BR activity in reproductive structures has remarkable potential for optimizing crop productivity, in some cases even in a post-harvest setting. This means that the dissection of the genetic and molecular pathways underlying BR responses during plant reproduction will greatly expand our biotechnological toolbox.

## Data Availability

No new datasets were generated or analyzed in this study.
